# Current developments in the Global Governance arena: where is alcohol headed?

**DOI:** 10.7189/jogh.09.020305

**Published:** 2019-12

**Authors:** Sally Casswell

**Affiliations:** SHORE & Whariki Research Centre, College of Health, Massey University, Auckland, New Zealand

“While less than half of the world’s adults have consumed alcohol in the last 12 months, the global burden of disease caused by its harmful use is enormous. Disturbingly, it exceeds those caused by many other risk factors and diseases high on the global health agenda.” *(Dr Tedros, Director General, World Health Organisation (WHO) Foreword, Global Status Report on Alcohol and Health, 2018 [**1**]).*

In 2016, alcohol resulted in 3 million deaths worldwide and 5% of disability-adjusted life years in that year. Mortality resulting from alcohol consumption is higher than that caused by diseases such as tuberculosis, HIV/AIDS and diabetes [1].

Alcohol is increasingly produced, distributed and marketed by transnational corporations. These have consolidated into very large entities; the amalgamation between Anheuser Busch and SABMiller created one of the leading consumer product corporations. ABInBev’s annual revenue of US$ 45.6 billion in 2017 put it ahead of half of the world’s countries in terms of their GDP. Major target markets for the transnational alcohol corporations (TNACs) are low and middle-income countries (LMICs) with expanding economies, youthful populations, increasing urbanisation, openness to privatisation and in which people either don’t drink alcohol or drink largely non-commercial alcohol. The consolidation of the TNACs provide enormous resources to develop supply lines and market products in new markets.

As governments around the world have sought to respond to the threats posed by harmful use of alcohol (estimated to cost about 1-2% of GDP [2]) the TNACs have positioned themselves as part of the solution, engaging directly with governments and in corporate social responsibility initiatives, including public private partnerships (PPPs) [3]. Considerable enthusiasm for PPPs is illustrated by goal 17 of the Sustainable Development Goals (SDGs), which specifically emphasises partnerships with private industry; this enthusiasm reflects a lack of success in dealing with intractable health problems and reductions in government funding. Notable examples of public private partnerships with alcohol industry include Heineken’s partnership with the Global Fund to assist the transmission of vaccines and Diageo’s with UNITAR to work on drink-driving initiatives. In keeping with such initiatives the TNACs are represented in the global governance arena, for example, in UN meetings on NCDs.

In the public health field there is considerable concern over the implications of this positioning of TNACs in the global governance arena. The framings and activities used by the TNACs to position themselves as part of the solution to alcohol harm and the nature of the conflict of interests posed by their reliance on heavy drinking are issues which lead to questions regarding their positioning within the global health environment.

## IDENTIFYING AND RESPONDING TO RISKS

The industry is active in researching the issues of most threat to their business and designing appropriate responses. Diageo informed its shareholders of the threat inherent in “failure to address perceived growth in anti-alcohol sentiment” (p.20) [4] and Heineken cautioned: “Alcohol remains under scrutiny in many markets. This may prompt regulators to take further measures limiting HEINEKEN’s freedom to operate, such as restrictions or bans on advertising and marketing, sponsorship, availability of products, and increased taxes and duties leading to lower revenues and profit” (p.21) [5]. In carrying out its analysis of stakeholder interests and business risks in 2015, Diageo identified alcohol harm, NCDs and marketing and retail as strong risks to its business.

Marketing is crucial to the TNACs’ market expansion through normalisation of drinking in different contexts in LMICs and recruitment of new cohorts of drinkers in mature markets. The TNACs were early adopters of digital marketing and alcohol marketing is pervasive in social media. The need to preserve their ability to market alcohol has led to a focus on the promotion of voluntary codes and self-monitoring mechanisms which have been shown to be ineffective [6] but provide an industry friendly alternative to regulation.

In relation to the identified threats of alcohol harm and alcohol as an NCD risk, the TNACs primarily respond by framing issues and developing corporate social responsibility (CSR) initiatives rather than outright denials of the harms. However, alcohol industry funded research and publications have attempted to throw doubt in some key areas and the evidence of alcohol’s carcinogenicity has proved particularly worthy of industry response. Common framings which direct attention away from industry practices are the minority status of heavy drinkers, personal responsibility and the benefits of ‘moderate’ alcohol use. Underage drinkers and drink driving are acknowledged as issues and responded to by corporate social responsibility (CSR) initiatives.

## CORPORATE CITIZENSHIP – PATHWAY TO INFLUENCE

CSR is a primary strategy to build corporate citizenship for the TNACs and divert attention away from evidence-based effective strategies [7]. Where CSR reduces environmental impact or improves the well-being of workers is to be welcomed, however, much CSR activity of the TNACs is focused on mitigating threats inherent in the product. An example of CSR is the promotion of “responsible drinking” programmes, for example in schools in societies with low youth drinking prevalence where the messages normalise drinking. A recent CSR initiative was the introduction of ABInBev’s Smart Drinking Goals. Pledging to spend US$ 1 billion dollars over ten years this initiative uses social marketing, labelling and programmes aiming to change social norms; it responds to market demand by expanding product range in low and no alcohol beer. These and similar examples are critiqued for the ambiguity of their messages and lack of focus on the cost-effective evidence based policies included in the ‘best buys’ of the United Nations (UN) system [8]. A key concern for public health is such CSR initiatives not only exclude best buys but are concurrent with opposition to them.

These industry strategies are working; as Diageo informs its shareholders: “Proactive, evidence based engagement to build trust and deepen our relationship and reputation with governments, industry and other stakeholders … have successfully mitigated threats and built momentum in our engagement with governments around the world to shape more balanced regulatory outcomes” (p.20) [4].

## INCREASING RECOGNITION OF ALCOHOL HARM AND ROLE OF INDUSTRY

While the alcohol TNACs retain a high profile in the global governance arena a number of factors have contributed to a more contested environment. Alcohol as one of four identified risk factors for non-communicable diseases (NCDs) has gradually assumed a more prominent position on the global health agenda. Alcohol also causes harm well beyond NCDs and quantification in the Global Burden of Disease and Injury studies has highlighted this [9] (although alcohol’s harm to others is not yet adequately reflected in the data). The framework provided by the SDGs has increased awareness of alcohol as an exacerbating factor for poverty and restraint on development; and a cause of traffic crash and injury and violence, including gendered violence.

**Figure:**
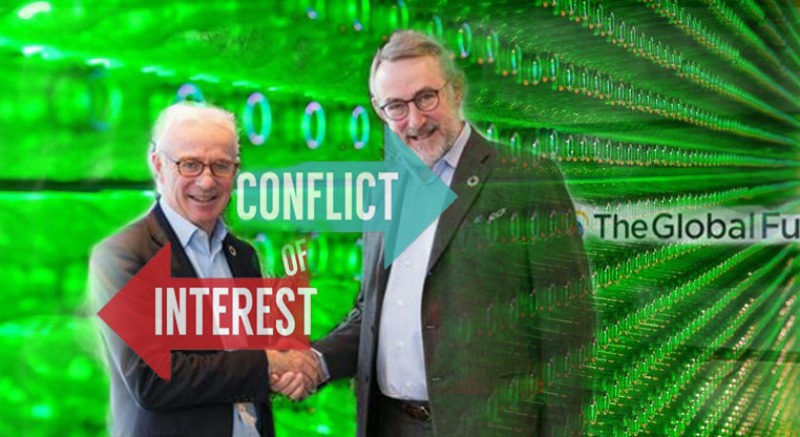
Photo: A partnership between Heineken and the Global Fund was strongly opposed by civil society and suspended after nine weeks. Source: IOGT press release, 9 March 2018 (used with permission).

The inherent conflict of interest and the consequent threat posed by alcohol industry interference in the policy arena contributes to the contestation. Data shows the TNACs rely on harmful use of alcohol for their profits. In cross country research, the proportion of alcohol sales consumed in harmful drinking occasions was about half in high income countries, 52% to 62% in middle income countries, and up to 90% in a province of South Africa [10]. Therefore the uptake of effective measures to reduce heavy drinking would impact significantly on sales and profits; the TNACs lobby to avoid implementation of effective policy especially alcohol taxation and marketing. The WHO Director General’s report to the Executive Board of the WHA in 2018 described interference by industry impeding the implementation of the “best buys” [11]. Similarly, the UN Interagency Task Force on NCDs highlighted pervasive industry attempts to influence government policy including industry briefing governments ahead of joint programming missions [11].

Governments of middle-income countries successfully targeted by the TNACs have also made their voices heard. Recent World Health Assemblies have heard requests from countries in Asia and Africa for enhanced support. In 2016, Botswana, supported by other African and Asian nations called upon the DG of WHO to “study the necessity and feasibility of a legally binding instrument to strengthen the public health response to harmful use of alcohol” [12].

## RESPONSE IN THE GLOBAL HEALTH GOVERNANCE ARENA

WHO is the major policy holder in response to alcohol harm, although the recent involvement of the UN via its focus on the NCD epidemic and the establishment of the SDGs has provided an important cross cutting response to harmful use of alcohol. The WHO response, after a long period of no attention, was in the form of the global strategy to reduce the harmful use of alcohol, endorsed by the WHA in 2010. However, few resources were committed [13], the global strategy is a list of potential strategies not a binding treaty and little effective impact has been seen particularly in LMICs.

A key issue in the UN context has been the lack of adequate response to conflict of interest. The WHO Framework for Engagement with Non State Actors is silent on alcohol. Calls for a Framework Convention on Alcohol Control (FCAC), which would crucially include an equivalent clause to 5.3 of the Framework Convention on Tobacco Control (FCTC), that limits tobacco industry involvement, were common in the years prior to the global strategy. Such calls died away after the global strategy endorsement; they have, however, begun to surface again, from academics, civil society and middle-income countries in the WHA.

The model provided by the FCTC seems particularly well suited to alcohol; while there are substantial differences between tobacco and alcohol in terms of the nature of the harms and the likely preferred end game, the similarities in business practices are substantial. There is no level of alcohol use completely without risk and the much publicised benefits to cardiovascular health of alcohol are as irrelevant to a policy response aiming to reduce harm as are the health benefits of tobacco.

Crucially, there is a need for an international treaty to respond to cross border promotion of alcohol, especially potent in the digital media, and the trade and economic treaties which privilege the corporations. The drivers of harmful use of alcohol and tobacco are the same: oversupply, marketing and affordability and an FCAC would look very similar to the FCTC with some additional requirements to address intoxication effects, for example, licensed hours of sale and preventing traffic crash [14].

## URGENT NEED FOR A GLOBAL RESPONSE TO REIN IN TNACS

The TNACs have no global and limited national restraints on their activities. Their expansion into the emerging markets of middle-income countries and reliance for profits on harmful alcohol use presents a clear and urgent threat to global health. As they report to their shareholders, they are currently succeeding in mitigating threats to their sales by subverting the development and implementation of effective alcohol policy. A global response to prevent marketing, over supply, inappropriate affordability and industry interference in policy is urgently required.

Whether the conditions that created the establishment of the FCTC, including a champion within the UN system, are likely to prevail in the current context is unclear. An FCAC is the TNACs’ worst nightmare and strong resistance from them and the member states which represent their interests is to be expected. However, the FCTC was once an unlikely aspiration and provides a valuable precedent for those concerned about the global health risk from the largely unregulated marketing and distribution of alcohol.
